# Prognostic and clinicopathological value of the prognostic nutritional index in prostate cancer treated with androgen deprivation therapy: a systematic review and meta-analysis

**DOI:** 10.3389/fonc.2026.1794606

**Published:** 2026-03-10

**Authors:** Huanli Huang, Jun Hu, Xingwei Lan, Hanxiao Li, Jingru Chen

**Affiliations:** 1 First Clinical College, Hubei University of Chinese Medicine, Wuhan, Hubei, China; 2 Department of Radiology, Hubei Provincial Hospital of Traditional Chinese Medicine, Affiliated Hospital of Hubei University of Chinese Medicine, Wuhan, Hubei, China; 3 School of Laboratory Medicine, Hubei University of Chinese Medicine, Wuhan, Hubei, China

**Keywords:** Androgen deprivation therapy, meta-analysis, overall survival, prognostic nutritional index, progression-free survival, prostate cancer

## Abstract

**Background:**

The prognostic nutritional index (PNI) has been associated with survival outcomes in multiple solid tumors, yet its prognostic relevance in prostate cancer patients undergoing androgen deprivation therapy (ADT)-based systemic treatment remains insufficiently characterized.

**Methods:**

We systematically searched PubMed, Embase, Cochrane Library, Web of Science, and China National Knowledge Infrastructure (CNKI) from database inception to September 11, 2025. Pooled hazard ratios (HRs) with 95% confidence intervals (CIs) were used to assess the association between PNI and survival outcomes; pooled odds ratios (ORs) with 95% CIs evaluated links with clinicopathological features. Subgroup analyses explored heterogeneity sources.

**Results:**

Ten retrospective studies involving 1, 847 patients were included. Low PNI was significantly associated with worse overall survival (HR = 2.082, 95% CI: 1.756–2.469, *p* < 0.001). Due to heterogeneous definitions of disease progression across studies, progression-related endpoints were analyzed separately by type. Low PNI consistently predicted inferior outcomes across multiple progression-free survival metrics: PFS (HR = 1.606, 95% CI: 1.328–1.942), radiographic PFS (rPFS; HR = 2.315, 95% CI: 1.525–3.514), and PSA-PFS (HR = 3.176, 95% CI: 2.169–4.652) (all *p* < 0.001). Subgroup analyses supported result robustness. Additionally, low PNI correlated significantly with Gleason score >7 (OR = 1.404, *p* = 0.018), bone metastasis (OR = 1.433, *p* = 0.015), LATITUDE high-risk status (OR = 1.898, p = 0.003), and CHAARTED-defined high tumor burden (OR = 1.950, *p* = 0.001), but not with age, visceral metastases, or EAU high-risk classification.

**Conclusions:**

In the context of ADT-based systemic therapy, low PNI is a significant predictor of poor survival and aggressive disease features, supporting its potential as a readily available biomarker for risk stratification and prognostic assessment in prostate cancer patients.

**Systematic review registration:**

https://www.crd.york.ac.uk/prospero/, identifier CRD420251145397.

## 1 Introduction

Prostate cancer (PCa) is one of the most prevalent malignancies in men worldwide, with its disease burden escalating due to population aging. According to GLOBOCAN 2022 estimates, PCa accounts for over 1.4 million new diagnoses and approximately 397, 000 deaths annually, ranking as the second most frequently diagnosed cancer in males (1). PCa exhibits marked biological and clinical heterogeneity. At diagnosis, the majority of patients present with localized or locally advanced hormone-sensitive prostate cancer (HSPC), which is initially responsive to androgen deprivation therapy (ADT) (2, 3). Although ADT-based regimens can control disease progression for a period, a subset of patients eventually experiences biochemical or radiographic progression, transitioning to castration-resistant prostate cancer (CRPC) with increased risk of recurrence and progression (4, 5). Recent guidelines and clinical studies indicate that ADT in combination with androgen receptor pathway inhibitors (ARPIs; such as abiraterone or enzalutamide) or combined with cytotoxic chemotherapy has become a standard first-line systemic treatment option for metastatic PCa, resulting in further improvements in overall survival (6–9). Nevertheless, therapeutic benefits vary substantially across individuals. Prognostic assessment based solely on traditional indicators, such as prostate-specific antigen (PSA) level, Gleason score, and tumor burden, is insufficient to fully capture this heterogeneity (10, 11). Therefore, identifying novel biomarkers that more accurately predict therapeutic efficacy and prognosis is of considerable clinical importance for optimizing treatment strategies and improving patient outcomes.

In recent years, systemic inflammation and nutritional status have been implicated in modulating tumor progression and treatment resistance by altering the tumor microenvironment (12). Several inflammation- and nutrition-related indices derived from peripheral blood parameters, such as systemic immune-inflammation index (SII) (13), controlling nutritional status (CONUT) score (14), geriatric nutritional risk index (GNRI) (15), and albumin-to-globulin ratio (AGR) (16), have been reported as potential prognostic serum biomarkers in various malignancies. Among these, the prognostic nutritional index (PNI), calculated using serum albumin and lymphocyte counts, has been validated as a marker associated with adverse outcomes in gastric, liver, and bladder cancers, underscoring its clinical utility as a simple, accessible indicator (17–19).

Previous meta-analyses examining the association between PNI and PCa outcomes have often pooled patients across diverse disease stages and treatment regimens, resulting in high clinical heterogeneity and limited comparability among study populations (20–22). Moreover, the definition of progression-free survival (PFS) varied across studies, such as radiological PFS (rPFS), PSA-PFS, and biochemical recurrence-free survival (BCR-PFS). Pooling these heterogeneous endpoints without stratification may increase methodological heterogeneity and undermine the clinical interpretability of the findings. Consequently, this systematic review and meta-analysis specifically focuses on patients receiving ADT-based systemic therapy. We aim to evaluate the independent prognostic value of baseline PNI in this context, stratify analyses by disease stage and treatment regimen, differentiate types of PFS endpoints, and explore associations between PNI and key clinicopathological features.

## 2 Materials and methods

### 2.1 Study guideline

This meta-analysis adhered to the Preferred Reporting Items for Systematic Reviews and Meta-Analyses (PRISMA) guidelines (23). The study protocol was prospectively registered in the International Prospective Register of Systematic Reviews (PROSPERO) under registration number CRD420251145397.

### 2.2 Search strategy

A systematic literature search was conducted in Embase, PubMed, the Cochrane Library, Web of Science, and China National Knowledge Infrastructure (CNKI) from the databases’ inception to September 11, 2025, without language restrictions. Search terms were constructed around two core concepts: “prognostic nutritional index” and “prostate cancer, “and were adapted according to the specific syntax of each database. For international databases, the search strategy was: (“prognostic nutritional index” OR “PNI”) AND (“prostate cancer” OR “prostatic carcinoma” OR “prostatic adenocarcinoma” OR “PCa”). To ensure comprehensiveness, treatment-related terms such as “ADT” or “endocrine therapy” were not included as mandatory search components. Instead, during the full-text screening phase, each potentially eligible study was individually reviewed to confirm that participants were receiving ADT-based systemic therapy. Additionally, reference lists of included studies and relevant reviews were manually screened for additional eligible studies.

### 2.3 Inclusion and exclusion criteria

Patients included should satisfy the following criteria (1): Patients with pathologically confirmed PCa who received ADT-based systemic therapy (2); Studies reporting the relationship between baseline PNI and survival outcomes (3); cutoff values should be identified to divide low/high PNI (4); hazard ratios (HRs) and associated 95% confidence intervals (CIs) of survival out comes should be available or calculable; and (5) available survival outcomes, such as OS, PFS, PSA-PFS, rPFS, or cancer-specific survival (CSS). Studies conforming to the following criteria were excluded (1): reviews, letters, conference abstracts, comments, or case reports (2); Duplicate publications or studies with overlapping patient cohorts (3); Animal experimental studies (4); Studies involving treatments not based on ADT.

### 2.4 Data extraction and quality assessment

Two reviewers (HHL and LXW) independently assessed and extracted data from the included studies, and two additional reviewers (HJ and CJR) verified the extracted information. Any discrepancies were resolved by a third adjudicator (LHX). Extracted information included: first author, publication year, country, sample size, patient age, study design, study period, disease stage, type of endocrine therapy, follow-up duration, PNI cutoff value and its determination method, survival endpoints, type of survival analysis, and HRs with 95% CI. Study quality was assessed using the Newcastle-Ottawa Scale (NOS), which evaluates three domains: selection of study participants, comparability of cohorts, and outcome assessment. The NOS scores range from 0 to 9, with studies scoring ≥ 6 considered to be of high quality (24).

### 2.5 Statistical analysis

The association between PNI and survival outcomes was evaluated using pooled HRs and 95% CIs. When both univariate and multivariate HRs were reported in the same study, the multivariable-adjusted HR was preferentially extracted. Between-study heterogeneity was assessed using Cochran’s Q test and the I² statistic. Heterogeneity was defined as *p* < 0.10 for the Q test and *I*² > 50%; a random-effects model was used in such cases, and a fixed-effects model otherwise. Given the anticipated clinical heterogeneity in disease setting and treatment intensity across cohorts, we additionally performed random-effects meta-analyses for all key survival outcomes as sensitivity analyses.

Subgroup analyses were conducted to explore potential sources of heterogeneity. Pooled odds ratios (ORs) and 95% CIs were calculated to examine the relationship between PNI and clinicopathological features. Publication bias was assessed using Begg’s funnel plot and Egger’s regression test. The trim and fill method was used to evaluate the impact of small study effects on the pooled effect estimate, and sensitivity analysis by sequentially removing one study at a time was performed to assess the robustness of the results. All analyses were conducted using Stata version 15.0 (StataCorp LP, College Station, Texas, USA) and R version 4.5.1 (R Core Team, Vienna, Austria), with a two-sided significance level of α = 0.05.

## 3 Results

### 3.1 Process of literature search

**Figure 1:**
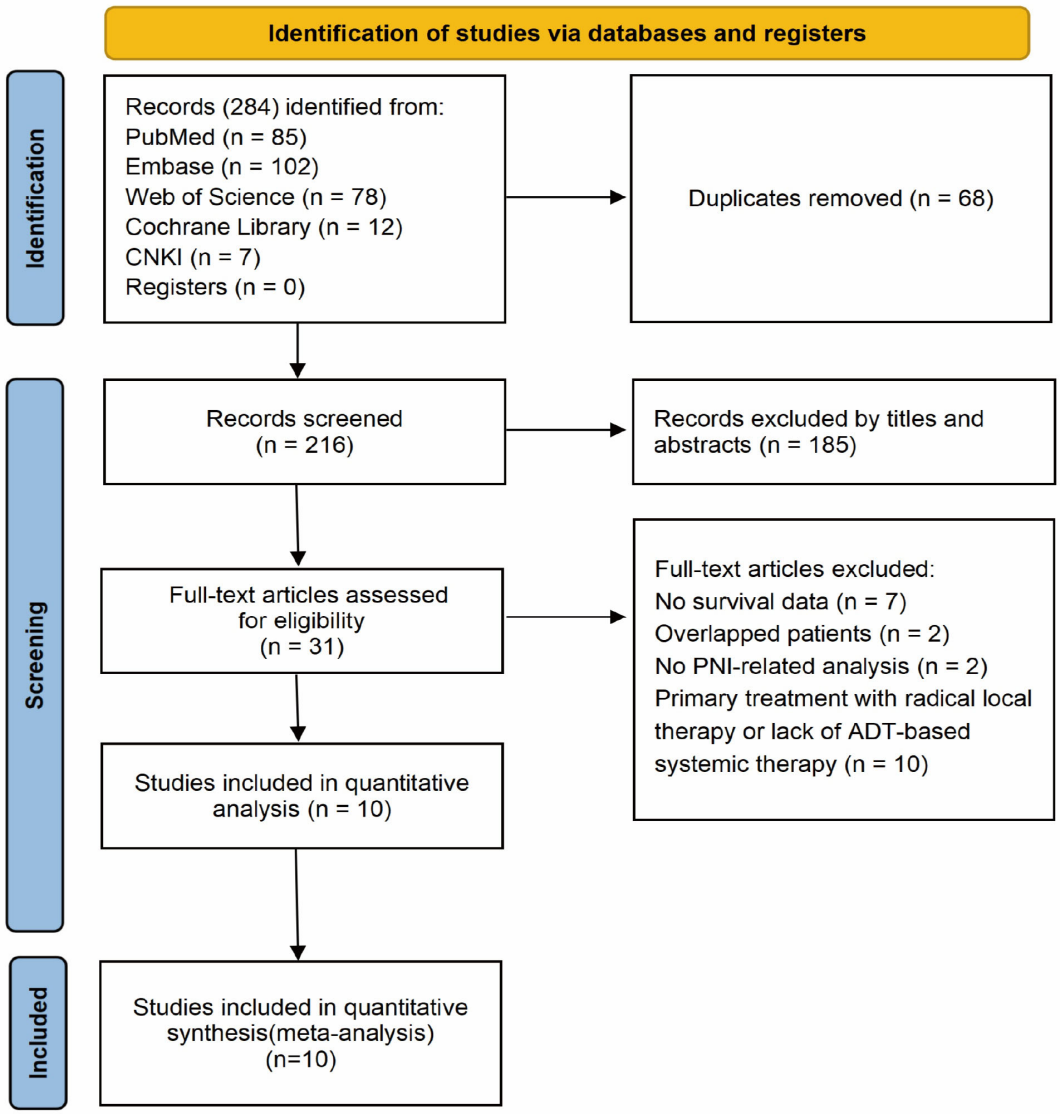
PRISMA flow chart of the data search.

A total of 284 records were initially identified. After duplicate removal, 216 unique records remained. Following title and abstract screening, 185 irrelevant records were excluded. The full texts of 31 articles were assessed for eligibility, of which 21 were excluded for the following reasons: primary treatment with radical local therapy or lack of ADT-based systemic therapy (n = 10), absence of usable survival data (n = 7), overlapping patient cohorts (n = 2), and no PNI-related analysis (n = 2). Ultimately, 10 studies comprising 1, 847 patients were included in the meta-analysis (22, 25–33) (**Figure 1**).

### 3.2 Characteristics of the included studies

**Table 1** summarizes the basic characteristics of the included studies (22, 25–33) published between 2017 and 2025. Five were conducted in China (27, 29, 31–33), four in Turkey (25, 26, 28, 30), and one in Japan (22); Seven were published in English (22, 25–30), and three in Chinese (31–33). The median sample size was 167 patients (range: 40–353). Four studies enrolled patients with mCRPC (26, 28, 29, 32), while six included patients with HSPC, of which four focused on metastatic HSPC (mHSPC) (22, 25, 30, 33), and two included mixed HSPC populations (non-metastatic and metastatic) (27, 31). The median PNI cut-off value was 48.3 (range: 40.8–50.5), and all studies determined their cut-off using receiver operating characteristic (ROC) curve analysis. Details of ROC cut-off derivation are summarized in **Supplementary Table 1**. All studies reported the association between PNI and OS (22, 25–33). Four reported PFS (22, 27, 31, 33), three reported rPFS and PSA-PFS (26, 29, 32), and three reported CSS (25, 27, 31). The NOS scores of the included studies ranged from 7 to 8 (**Supplementary Table 2**).

**Table 1:** Basic characteristics of included studies in this meta-analysis.

Study	Year	Country	Sample size	Age(years)	Study design	Study duration	Disease	Therapy type	Follow-up (months) median(range)	Cut-off value	Cut-off determination	Survival endpoint	Survival analysis	NOS score
Wang et al. (31)	2017	China	290	75(67-79)	Retrospective	2010-2014	HSPC	ADT	37(24-50.3)	50.25	ROC curve	OS, CSS, PFS	Multivariate	8
Fan et al. (29)	2017	China	112	72 (66–77)	Retrospective	2012–2016	mCRPC	ADT+ ARPIs	22.2 (20.3–24.1)	50.5	ROC curve	OS, rPFS, PSA-PFS	Multivariate	7
Li et al. (27)	2020	China	280	76 (67.25–79)	Retrospective	2013–2016	HSPC	ADT	46.0 (31.0–59.0)	50.2	ROC curve	OS, CSS, PFS	Multivariate	8
Küçükarda et al. (34)	2021	Turkey	101	71 (64.5–76)	Retrospective	2012–2019	mCRPC	ADT+ ARPIs	13.5 (6.8–21.4)	46.62	ROC curve	OS, rPFS, PSA-PFS	Multivariate	7
Sun et al. (32)	2022	China	40	70.21 ± 8.36	Retrospective	2017–2021	mCRPC	ADT+ ARPIs	19.7(10.1-26.5)	48.3	ROC curve	OS, rPFS, PSA-PFS	Multivariate	7
Ma et al. (33)	2022	China	97	67 (46–86)	Retrospective	2015–2018	mHSPC	ADT	39(4-75)	48.3	ROC curve	OS, PFS	Multivariate	8
Ellez et al. (25)	2023	Turkey	108	68.54 (61.05–74.19)	Retrospective	2010–2021	mCSPC	ADT	NR	49.75	ROC curve	OS, CSS	Multivariate	8
Yamada et al. (22)	2023	Japan	353	73 (46–93)	Retrospective	2000–2019	mHSPC	ADT	48.53 (0.6–210.1)	47.71	ROC curve	OS, PFS	Multivariate	8
Taban et al. (28)	2025	Turkey	299	65.2 ± 9.0	Retrospective	2011–2020	mCRPC	ADT+ ARPIs	18.9 (9.3–30.2)	40.8	ROC curve	OS	Multivariate	8
Hacioglu et al. (30)	2025	Turkey	167	73 (67–77)	Retrospective	2019–2024	mHSPC	ADT+ ARPIs	18.3 (12.9–25.1)	49.98	ROC curve	OS	Multivariate	7

Age is reported as median (range) or mean ± SD, according to the original study, HSPC hormone-sensitive prostate cancer, mCRPC metastatic castration-resistant prostate cancer, mHSPC metastatic hormone-sensitive prostate cancer, mCSPC metastatic castration-sensitive prostate cancer (considered equivalent to mHSPC and grouped with mHSPC in subgroup analyses), Therapy type was categorized as ADT (± chemotherapy) or ADT + ARPIs (± chemotherapy), ADT androgen deprivation therapy, ARPIs androgen receptor pathway inhibitors, NR not reported, ROC receiver operating characteristics, OS overall survival, CSS cancer-specific survival, PFS progression-free survival, rPFS radiographic progression-free survival, PSA-PFS prostate-specific antigen progression-free survival, NOS Newcastle-ottawa scale.

### 3.3 PNI and OS

**Figure 2:**
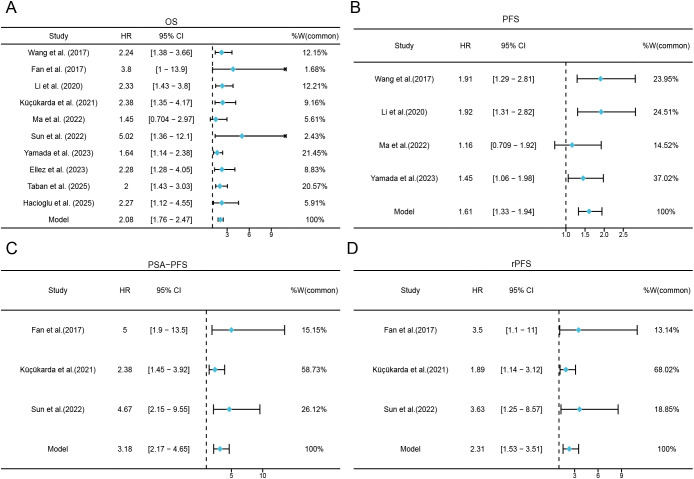
Forest plots for the association between PNI and survival outcomes. **(A)** Forest plots for the association between PNI and OS; **(B)** Forest plots for the association between PNI and PFS; **(C)** Forest plots for the association between PNI and PSA-PFS; **(D)** Forest plots for the association between PNI and rPFS.

Ten studies (1, 847 patients) (22, 25–33) evaluated the association between baseline PNI and OS. Between-study heterogeneity was negligible (*I*² = 0%, *p* = 0.680), prompting the use of a fixed-effects model. Random-effects sensitivity analysis yielded an identical pooled estimate (HR = 2.082, 95% CI: 1.756–2.469; **Supplementary Table 3**), indicating that the association is robust to model specification and may be interpreted as an average effect across clinically heterogeneous cohorts. As shown in **Figure 2A** and **Table 2**, the pooled HR was 2.082 (95% CI: 1.756–2.469, *p* < 0.001), indicating that a lower PNI was significantly associated with poorer OS.

**Table 2:** Subgroup analysis of prognostic role of PNI for overall survival and other clinical outcomes in patients with prostate cancer.

Subgroup	No. of studies	No. of patients	HR (95% CI)	*p*	Effect model	Heterogeneity	
						*I*^2^(%)	Ph
Panel A. Overall survival							
Total	10	1847	2.082(1.756, 2.469)	< 0.001	Fixed	0	0.680
Country							
China	5	819	2.300(1.718, 3.080)	< 0.001	Fixed	2.7	0.391
Other	5	1028	1.977(1.603, 2.439)	< 0.001	Fixed	0	0.773
Sample size							
≤140	5	458	2.332(1.687, 3.224)	< 0.001	Fixed	2.6	0.392
>140	5	1389	1.993(1.631, 2.436)	< 0.001	Fixed	0	0.767
Cut-off value							
≤48.3	5	890	1.926(1.543, 2.404)	< 0.001	Fixed	17.7	0.302
>48.3	5	957	2.331(1.785, 3.044)	< 0.001	Fixed	0	0.967
Disease							
mHSPC	4	725	1.811(1.391, 2.357)	< 0.001	Fixed	0	0.644
mCRPC	4	552	2.312(1.725, 3.099)	< 0.001	Fixed	1.9	0.383
HSPC	2	570	2.287(1.619, 3.230)	< 0.001	Fixed	0	0.913
Treatment							
ADT	5	1128	1.946(1.562, 2.424)	< 0.001	Fixed	0	0.624
ADT + ARPIs	5	719	2.306(1.760, 3.022)	< 0.001	Fixed	0	0.548
Panel B. Progression-related endpoints							
PFS	4	1020	1.606(1.328, 1.942)	< 0.001	Fixed	16.5	0.309
rPFS	3	253	2.315(1.525, 3.514)	< 0.001	Fixed	0	0.375
PSA-PFS	3	253	3.176(2.169, 4.652)	< 0.001	Fixed	36.1	0.209
Panel C. Cancer-specific survival (CSS)							
CSS	3	678	2.507(1.812, 3.469)	<0.001	Fixed	0	0.764

HSPC hormone-sensitive prostate cancer, mCRPC metastatic castration-resistant prostate cancer, mHSPC metastatic hormone-sensitive prostate cancer, ADT androgen deprivation therapy, ARPIs androgen receptor pathway inhibitors, OS overall survival.

Subgroup analyses stratified by country, sample size, PNI cut-off value, disease stage, and treatment type (**Table 2**) consistently showed that decreased PNI remained a significant predictor of worse OS across all subgroups (all *p* < 0.05).

### 3.4 PNI and PFS

Seven studies involving 1, 273 patients (22, 26, 27, 29, 31–33) reported progression-related outcomes, but endpoint definitions varied across studies. Therefore, we synthesized progression outcomes separately by endpoint type (**Table 2**). Low PNI was associated with shorter PFS (HR = 1.606, 95% CI: 1.328–1.942, *p* < 0.001) (**Figure 2B**). Similarly, low PNI predicted worse rPFS (HR = 2.315, 95% CI: 1.525–3.514, *p* < 0.001) (**Figure 2D**). Consistent results were observed for PSA-PFS (HR = 3.176, 95% CI: 2.169–4.652, *p* < 0.001) (**Figure 2C**). Random-effects sensitivity analyses produced materially similar pooled estimates for each endpoint (**Supplementary Table 3**).

### 3.5 PNI and CSS

Three studies comprising 678 patients (25, 27, 31) investigated the relationship between PNI and CSS. Low PNI was independently associated with worse CSS (HR = 2.507, 95% CI: 1.812–3.469, *p* < 0.001) (**Table 2**), suggesting that reduced baseline PNI is linked to an increased risk of prostate cancer-specific mortality.

### 3.6 Association between PNI and clinicopathological features

Four studies involving 909 patients (27, 28, 31, 32) reported data on PNI and clinicopathological characteristics. The analyzed variables included age (≥70 years vs <70 years), Gleason score (>7 vs ≤7), bone metastasis (yes vs no), visceral metastasis (yes vs no), European Association of Urology (EAU) risk stratification (high-risk vs low/intermediate-risk), LATITUDE criteria (high-risk vs low-risk), and CHAARTED criteria (high-volume vs low-volume disease).

As shown in **Table 3**; **Supplementary Figure 1**, low PNI was significantly associated with more aggressive disease features, including higher Gleason score (OR = 1.404, 95% CI: 1.059–1.862, *p* = 0.018) and presence of bone metastasis (OR = 1.433, 95% CI: 1.074–1.913, *p* = 0.015). In addition, low PNI was significantly correlated with high-risk/high-volume disease according to the LATITUDE criteria (OR = 1.898, 95% CI: 1.239–2.908, *p* = 0.003) and the CHAARTED criteria (OR = 1.950, 95% CI: 1.311–2.901, *p* = 0.001).

**Table 3:** Association between PNI and clinicopathological features in patients with prostate cancer.

Variables	No. of studies	No. of patients	OR (95% CI)	*p*	Effect model	Heterogeneity	
						*I*^2^(%)	ph
Age (≥70 vs <70)	2	407	1.284(0.868, 1.900)	0.210	Fixed	0	0.459
Gleason score (>7 vs ≤7)	4	909	1.404(1.059, 1.862)	0.018	Fixed	39.3	0.176
Bone metastasis (yes vs no)	4	909	1.433(1.074, 1.913)	0.015	Fixed	0	0.944
Visceral metastasis (yes vs no)	2	652	1.251(0.813, 1.927)	0.308	Random	78.4	0.032
EAU risk stratification (high vs low/intermediate)	2	570	1.107(0.643, 1.906)	0.713	Fixed	0	0.8
LATITUDE criteria (high vs low risk)	2	461	1.898(1.239, 2.908)	0.003	Random	69.9	0.068
CHAARTED criteria (high vs low volume)	2	461	1.950(1.311, 2.901)	0.001	Fixed	13.1	0.623

EAU European Association of Urology, LATITUDE high-risk definition derived from the LATITUDE trial, CHAARTED high-volume definition derived from the CHAARTED trial.

However, no significant associations were observed between PNI and age (OR = 1.284, 95% CI: 0.868–1.900, *p* = 0.210), visceral metastasis (OR = 1.251, 95% CI: 0.813–1.927, *p* = 0.308), or EAU-ESTRO SIOG high-risk stratification (OR = 1.107, 95% CI: 0.643–1.906, *p* = 0.713) (**Supplementary Figure 1**).

### 3.7 Publication bias

**Figure 3:**
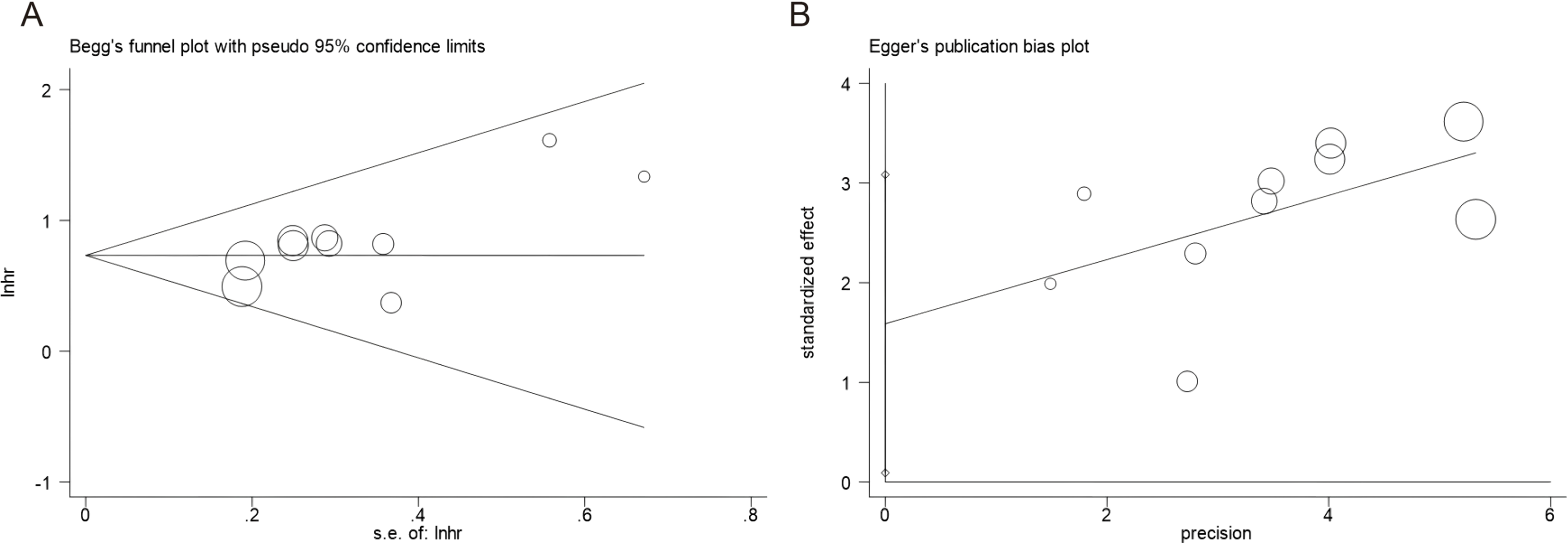
Publication bias test by using Begg’s test and Egger’s test. **(A)** Begg’s test for OS, *p* = 0.128; **(B)** Egger’s test for OS, *p* = 0.109.

Potential publication bias was evaluated using Begg’s test and Egger’s test. Visual inspection of the funnel plots revealed no marked asymmetry (**Figure 3**). Statistically, no significant publication bias was detected: for OS, Begg’s test *p* = 0.128 and Egger’s test *p* = 0.109.

### 3.8 Trim-and-fill and sensitivity analysis

**Figure 4:**
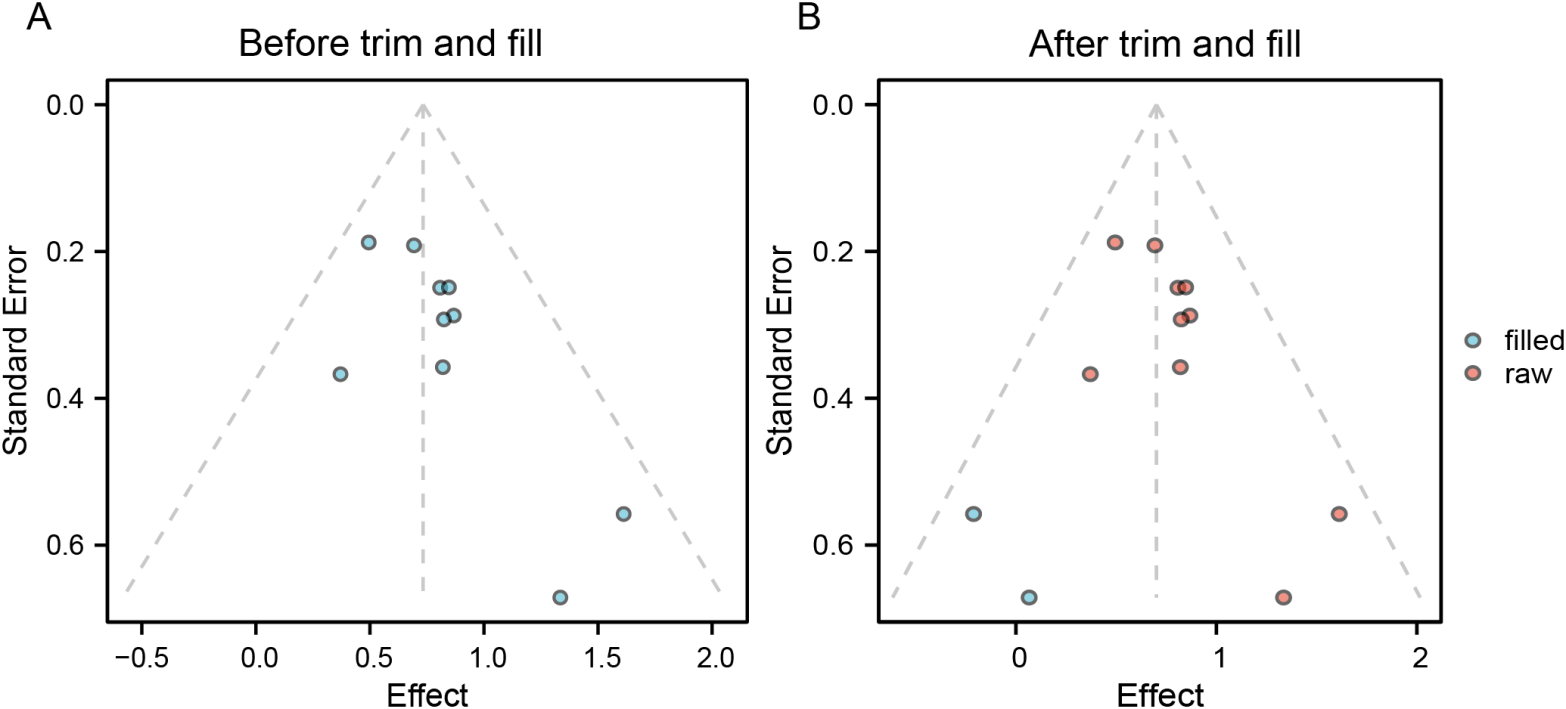
Distribution of effect sizes before and after trim-and-fill analysis. **(A)** Before trim and fill; **(B)** After trim and fill.

Duval and Tweedie’s trim-and-fill method was used to assess the influence of small-study effects. For OS (**Figure 4**), two studies were imputed, yielding an adjusted pooled HR of 2.015 (95% CI: 1.705–2.381, *p* < 0.001). The direction and statistical significance of the effect estimates remained consistent before and after adjustment.

A leave-one-out sensitivity analysis showed that the pooled HR for OS ranged from 2.037 to 2.222. after sequentially excluding each individual study; all results remained statistically significant (**Supplementary Figure 2**). These findings indicate that the main conclusions of this meta-analysis are not driven by any single study.

## 4 Discussion

In this systematic review and meta-analysis of 10 retrospective studies involving 1, 847 patients, we evaluated the prognostic and clinicopathological significance of baseline PNI in PCa patients receiving ADT-based systemic treatment. A lower PNI was significantly associated with shorter OS and poorer progression-related outcomes across endpoint definitions (PFS, rPFS, and PSA-PFS). Analysis of CSS further indicated that low PNI was linked to an increased risk of prostate cancer–specific mortality. The prognostic value of PNI remained consistent across all predefined subgroups. Moreover, low PNI was significantly associated with aggressive disease features, including Gleason score >7, bone metastasis, high-risk disease according to LATITUDE criteria, and high-volume disease according to CHAARTED criteria, suggesting that patients with reduced PNI tend to present with a more aggressive tumor phenotype.

This study also underscores the importance of selecting objective endpoints in prognostic research. In our analyses, rPFS yielded the lowest between-study heterogeneity (I² = 0%) compared with PSA-PFS (I² = 36.1%) and conventional PFS (I² = 16.5%), supporting the greater reliability and reproducibility of objective, imaging-based endpoints. Importantly, we did not pool these progression definitions together; instead, PFS, rPFS, and PSA-PFS were synthesized separately to improve clinical interpretability. By contrast, PSA-PFS was associated with higher heterogeneity, likely due to inter-study variations in the definition of PSA progression and the susceptibility of PSA levels to non-neoplastic influences, including systemic inflammation, concomitant medications, and other non-tumor-related factors (35). Traditional PFS may also be affected by variability in assessment schedules and criteria and by event attribution in routine practice, which can increase measurement noise and between-study inconsistency. Nevertheless, these findings should be interpreted with caution given the limited number of eligible studies and the relatively small pooled sample size, which may reduce statistical power and constrain the generalizability of our conclusions.

From a clinical perspective, PNI is attractive because it is practical. It is derived from routine laboratory parameters that are available before treatment initiation and does not require specialized testing. The current evidence does not support a single universally actionable cut-off. Nonetheless, lower PNI values may indicate reduced host reserve and help identify patients who warrant closer monitoring and earlier supportive-care assessment during ADT-based systemic therapy.

Several prior studies have supported the independent prognostic value of PNI in PCa across different settings. In elderly patients, a favorable nutritional status defined by PNI was an independent protective factor for PSA-PFS and PFS, and integrating PNI into conventional prognostic models, such as AJCC staging, significantly enhanced discriminative ability (36). Among patients undergoing radical prostatectomy, preoperative low PNI was associated with increased biochemical recurrence risk and worse BCR-PFS, remaining significant in multivariate analyses (37). Further research suggested that combining PNI with clinicopathological variables may improve postoperative risk stratification and follow-up planning (38). These data support the relevance of host status across the disease continuum. In contrast, comparative studies evaluating PNI alongside other inflammation- and nutrition-related indices in PCa remain limited and show inconsistent results (39, 40). For example, Li et al. (36) found that the Nutritional Risk Index (NRI) had a higher C-index than PNI and CONUT for predicting PSA-PFS and PFS, and that CONUT most inaccurately predicted nutritional status classification. Similar comparisons have also been reported in other tumor types; for instance, in advanced oral squamous cell carcinoma, PNI showed better discrimination than SII for recurrence prediction (AUC = 0.72) (41). However, evidence in ADT-treated PCa cohorts is sparse and heterogeneous, which limits robust head-to-head comparisons within the same patient population and prevents clear conclusions about superiority or incremental value.

Several biological mechanisms may plausibly link low PNI to poor outcomes under ADT-based systemic therapy. PNI, originally proposed by Buzby et al. (42), integrates serum albumin and peripheral lymphocyte count and provides a compact assessment of nutritional status and immune function. Albumin reflects nutritional status but also functions as a negative acute-phase reactant, and low albumin often indicates sustained inflammatory burden and illness severity rather than reduced intake alone (43). Peripheral lymphocyte count serves as a marker of cellular immune competence, and lymphopenia may indicate impaired anti-tumor immune surveillance (44). Preclinical data suggest that inflammatory signaling, including an IL-6/STAT3 axis, can upregulate AR signaling and AR splice variants such as AR-V7, which has been implicated in resistance to AR-targeted therapy (45, 46). In parallel, lymphopenia may reflect impaired immune surveillance. Natural killer (NK) cells play a key role in eliminating stressed or senescent tumor cells, and reduced lymphocyte/NK-cell competence could therefore allow treatment-tolerant cell populations to persist and expand under therapeutic pressure (47). Consistent with these concepts, the elevated hazard ratio observed in the mCRPC subgroup (**Table 2**) suggests that host nutritional and immune dysregulation may exert a stronger adverse impact in advanced disease. Many patients with advanced cancer develop cancer-related cachexia and systemic inflammation, which are characterized by pro-inflammatory cytokine activity (e.g., TNF-α) and activation of catabolic pathways that drive muscle wasting and metabolic dysfunction (48, 49). In patients receiving ADT-based systemic therapy, long-term androgen deprivation and toxicities from androgen receptor pathway inhibitors may further worsen appetite, body composition, and inflammatory status, thereby progressively compromising host reserve (50, 51). This constellation may reduce treatment tolerance and the ability to sustain treatment intensity, increasing vulnerability to toxicity and interruptions in treatment delivery (52–54). Together, these considerations provide a biologically plausible rationale for the association between low PNI and inferior outcomes in ADT-treated cohorts.

The clinicopathological correlations observed in our analysis are consistent with this framework. Patients with low PNI had higher proportions of Gleason score >7 and bone metastasis, suggesting that higher-grade and more disseminated disease may coexist with greater nutritional and immune compromise. The significant association between low PNI and LATITUDE-defined high-risk disease and CHAARTED-defined high-volume disease, but not with EAU high-risk stratification (**Table 3**), may reflect differences in target populations and in what these tools capture. LATITUDE and CHAARTED cohorts predominantly include patients with high tumor burden, in whom systemic depletion and inflammation are more common and may be better reflected by PNI (55). Accordingly, within CHAARTED-defined high-volume patients, low PNI may help identify a subgroup with particularly poor prognosis who could be prioritized for multidisciplinary assessment and individualized discussion of treatment intensification when clinically eligible (56). In contrast, EAU risk stratification encompasses a broader spectrum from localized to metastatic disease (57). These associations suggest that PNI may complement tumor-centric risk tools by capturing host reserve and systemic inflammation.

The prognostic utility of PNI has also been corroborated in meta-analyses across multiple solid tumors, in which low PNI is generally associated with poorer survival outcomes and more advanced disease features (58–62). These convergent observations support the broader relevance of host nutritional and immune status in cancer prognosis.

Several limitations of this meta-analysis should be acknowledged. First, all included studies were retrospective cohort designs, which may introduce selection bias and residual confounding. While all studies reported multivariable-adjusted hazard ratios, adjustment variables varied across studies. Key unmeasured factors include comorbidities, acute infections, performance status, and treatment selection. We assessed robustness using leave-one-out sensitivity analyses and small-study effect tests (Begg’s and Egger’s tests, trim-and-fill), which did not materially alter pooled estimates; however, these tests have limited power with few included studies. Second, we could not assess dynamic PNI changes during ADT or correlate on-treatment PNI trajectories with treatment response. Most studies reported only baseline PNI, with serial measurements either unavailable or measured at non-comparable time points. Future prospective studies should standardize longitudinal PNI monitoring to determine whether PNI trajectories offer prognostic value beyond baseline assessment. Third, no universally accepted PNI cut-off exists. Although subgroup analyses suggest robustness across thresholds, lack of standardization hinders direct comparability and clinical implementation. Fourth, all included studies originated from East Asian and Middle Eastern populations, limiting generalizability to other ethnic groups. Large-scale, multicenter prospective trials across diverse cohorts are needed to externally validate PNI’s prognostic role in endocrine-treated PCa.

Future research should explore the incremental value of PNI when integrated with established clinical risk frameworks and other host-related biomarkers. Such work should perform direct head-to-head comparisons within the same ADT-treated cohorts using standardized endpoints and unified multivariable models. Interventional studies are needed to test whether strategies that improve nutritional and inflammatory status can translate into better treatment tolerance and clinical outcomes. These strategies may help preserve muscle mass, reduce systemic inflammation, and stabilize albumin and lymphocyte counts, thereby supporting functional status and treatment delivery. However, whether improving PNI translates into survival benefit requires prospective validation.

## 5 Conclusion

In summary, within the context of ADT-based systemic treatment, PNI serves as a robust and independent predictor of adverse survival outcomes. Patients with decreased PNI are more likely to exhibit aggressive disease characteristics, including higher Gleason score, bone metastasis, and high-risk/high-volume disease as defined by the LATITUDE and CHAARTED criteria. As a simple, efficient, and reproducible biomarker derived from routine blood tests, PNI demonstrates significant potential as a practical prognostic indicator for this patient population.

## Data Availability

The original contributions presented in the study are included in the article/**Supplementary Material**. Further inquiries can be directed to the corresponding authors.
