# Supplementary material for: Prognostic and clinicopathological value of the prognostic nutritional index in prostate cancer treated with androgen deprivation therapy: a systematic review and meta-analysis
Source: Front Oncol. 2026 Mar 10;16:1794606. doi: 10.3389/fonc.2026.1794606 (PMC13008628; doi:10.3389/fonc.2026.1794606)
Supplement: Supplementary file 1 [file DataSheet1.docx]

**SUPPLEMENTAL MATERIAL**

**Prognostic and clinicopathological value of the prognostic nutritional index in prostate cancer treated with androgen deprivation therapy: a systematic review and meta-analysis**

**Contents:**

• **Supplementary Table 1 ROC-based determination of PNI cut-off values in the included studies.**

• **Supplementary Table 2 Quality evaluation of the eligible studies with Newcastle–Ottawa Scale.**

**• Supplementary Table 3 Sensitivity analyses comparing fixed-effect and random-effects models.**

• **Supplementary Figure 1 Forest plots of association between PNI and clinicopathological factors.**

• **Supplementary Figure 2 Sensitivity analysis using the leave-one-out method for OS.**

**Supplementary Table 1 ROC-based determination of PNI cut-off values in the included studies.**

| **Study** | **Cut-off value** | **Baseline PNI timing** | **ROC endpoint** | **Cut-off selection rule** | **ROC performance** | **Notes** |
| --- | --- | --- | --- | --- | --- | --- |
| Wang et al. | 50.25 | pre-treatment | CSS | Maximized Youden index | Sensitivity: 53.0%, Specificity: 71.4%, AUC: 0.644, Youden index: 0.244 |  |
| Fan et al. | 50.5 | pre-treatment | OS | NR |  |  |
| Li et al. | 50.2 | before the prostate biopsy | CSS | NR |  |  |
| Küçükarda et al. | 46.62 | pre-treatment | NR | NR | AUC: 0.243 (p = 0.001) |  |
| Sun et al. | 48.3 | pre-treatment | Initial response to AA treatment | NR | AUC (95% CI) : 0.717 (0.610–0.824), Sensitivity:85.7%,Specificity:44.4%. |  |
| Ma et al. | 48.3 | pre-treatment | NR | NR |  |  |
| Ellez et al. | 49.75 | before prostate biopsy | CSS | NR |  |  |
| Yamada et al. | 47.71 | pre-treatment | OS | NR | AUC：0.61731 |  |
| Taban et al. | 40.8 | pre-treatment | NR | Median used for grouping (ROC-derived cut-off reported: 41.26) | Sensitivity: 59.1%, Specificity 59.7%, AUC: 0.643. | authors used median because ROC cut-off was close to median and no threshold with both high Se/Sp was identified |
| Hacioglu et al. | 49.98 | pre-treatment | OS | optimal balance between sensitivity and specificity | AUC: 0.288 (*p* = 0.001) |  |

PNI: Prognostic nutritional index, ROC: Receiver operating characteristic, OS: Overall survival, CSS: Cancer-specific survival, AUC: Area under the curve, NR; Not reported, AA: Abiraterone acetate. When the cut-off selection rule or ROC endpoint was not explicitly stated in the original publication, it was recorded as NR. For Taban et al., a ROC-derived cut-off (41.26) was reported, but the cohort median (40.8) was ultimately used for patient stratification.

**Supplementary Table 2 Quality evaluation of the eligible studies with Newcastle–Ottawa Scale.**

| author | **Selection(1-4)** | | | | **Comparability(1-2)** | | **Outcome(1-3)** | | | **score** |
| --- | --- | --- | --- | --- | --- | --- | --- | --- | --- | --- |
|  | Sel1 | Sel2 | Sel3 | Sel4 | Com1 | Com2 | Out1 | Out2 | Out3 |  |
| Wang et al.(2017) |  | a ★ | a ★ | a ★ | a ★ | b★ | b ★ | a ★ | b ★ | 8 |
| Fan et al.(2017) |  | a ★ | a ★ | a ★ | a ★ | b★ | b ★ | a ★ |  | 7 |
| Li et al.(2020) |  | a ★ | a ★ | a ★ | a ★ | b★ | b ★ | a ★ | b ★ | 8 |
| Küçükarda et al.(2021) |  | a ★ | a ★ | a ★ | a ★ | b★ | b ★ | a ★ |  | 7 |
| Sun et al.(2022) |  | a ★ | a ★ | a ★ | a ★ | b★ | b ★ | a ★ |  | 7 |
| Ma et al.(2022) |  | a ★ | a ★ | a ★ | a ★ | b★ | b ★ | a ★ | b ★ | 8 |
| Ellez et al.(2023) |  | a ★ | a ★ | a ★ | a ★ | b★ | b ★ | a ★ | b ★ | 8 |
| Yamada et al.(2023) |  | a ★ | a ★ | a ★ | a ★ | b★ | b ★ | a ★ | b ★ | 8 |
| Taban et al.(2025) | b ★ | a ★ | a ★ | a ★ | a ★ | b★ | b ★ | a ★ |  | 8 |
| Hacioglu et al.(2025) |  | a ★ | a ★ | a ★ | a ★ | b★ | b ★ | a ★ |  | 7 |

Sel1: Representativeness of the exposed cohort, Sel2: Selection of the non exposed cohort, Sel3: Ascertainment of exposure, Sel4: Demonstration that outcome of interest was not present at start of study, Com1: Comparability of cohorts on the basis of the design or analysis, Com2: study controls for any additional factor, Out1: Assessment of outcome, Out2: Was follow-up long enough for outcomes to occur, Out3: Adequacy of follow up of cohorts.

**Supplementary Table 3 Sensitivity analyses comparing fixed-effect and random-effects models.**

|  |  |  |  |  |  | Heterogeneity | |  |  |  |  |  |  |  | Heterogeneity | |
| --- | --- | --- | --- | --- | --- | --- | --- | --- | --- | --- | --- | --- | --- | --- | --- | --- |
| Outcome Subgroup | No.of studies | No.of patients | HR(95%CI) | *P* | Effect model | *I*² (%) | ph |  | Outcome Subgroup | No.of studies | No.of patients | HR(95%CI) | *P* | Effect model | *I*² (%) | ph |
| OS | 10 | 1847 | 2.082(1.756,2.469) | ＜0.001 | Fixed | 0 | 0.68 |  | OS | 10 | 1847 | 2.082(1.756,2.469) | ＜0.001 | Random | 0 | 0.68 |
| PFS | 4 | 1020 | 1.606(1.328,1.942) | ＜0.001 | Fixed | 16.5 | 0.309 |  | PFS | 4 | 1020 | 1.605(1.301,1.981) | ＜0.001 | Random | 16.5 | 0.309 |
| rPFS | 3 | 253 | 2.315(1.525,3.514) | ＜0.001 | Fixed | 0 | 0.375 |  | rPFS | 3 | 253 | 2.315(1.525,3.514) | ＜0.001 | Random | 0 | 0.375 |
| PSA-PFS | 3 | 253 | 3.176(2.169,4.652) | ＜0.001 | Fixed | 36.1 | 0.209 |  | PSA-PFS | 3 | 253 | 3.419(2.051,5.700) | ＜0.001 | Random | 36.1 | 0.209 |
| CSS | 3 | 678 | 2.507(1.812,3.469) | ＜0.001 | Fixed | 0 | 0.764 |  | CSS | 3 | 678 | 2.507(1.812,3.469) | ＜0.001 | Random | 0 | 0.764 |

OS: Overall survival, PFS: Progression-free survival, rPFS: Radiographic progression-free survival, PSA-PFS: Prostate-specific antigen progression-free survival, CSS: cancer-specific survival.

**Supplementary Figure 1** **Forest plots of association between PNI and clinicopathological factors.**

**
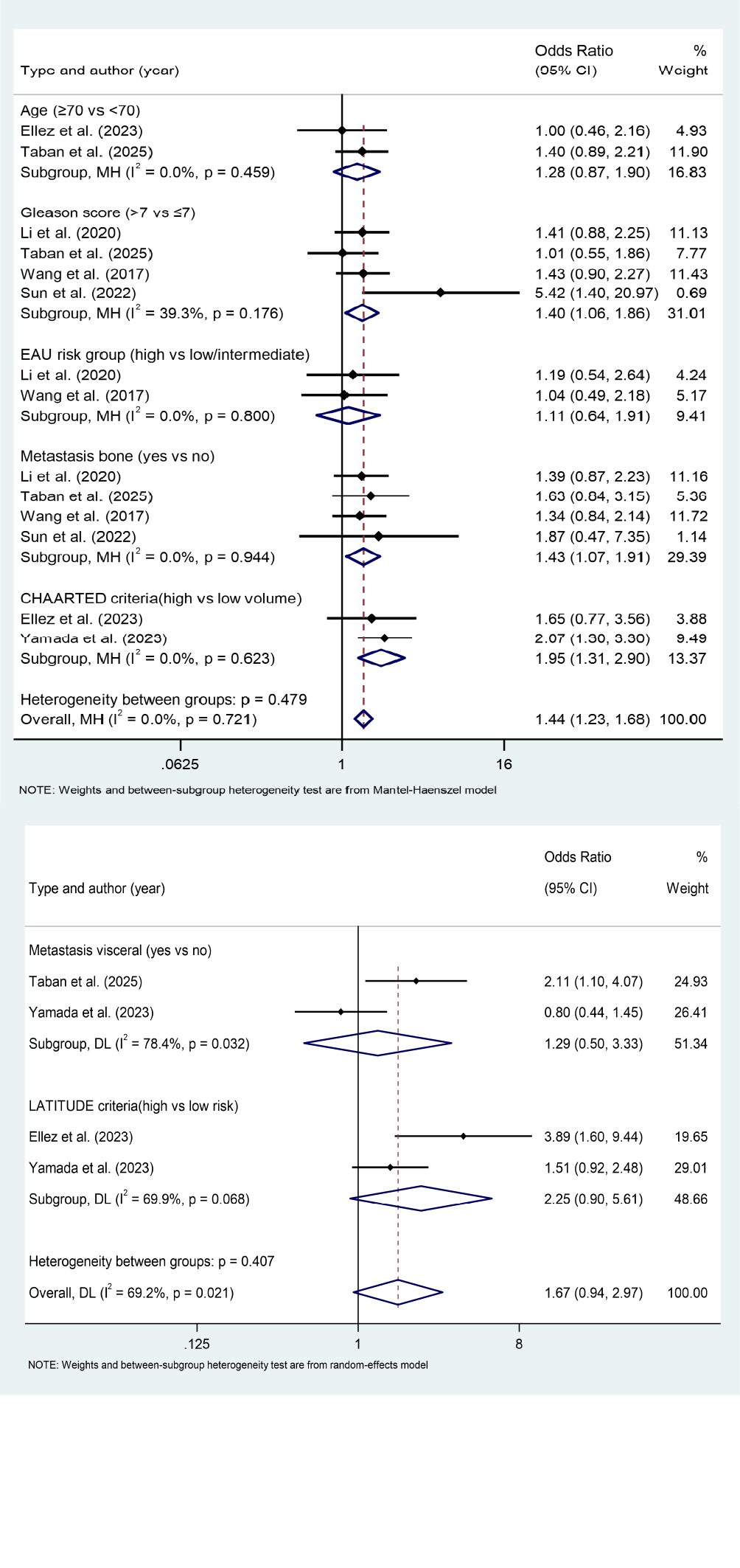
**

**Supplementary Figure 2 Sensitivity analysis using the leave-one-out method for OS.**
